# Investigating the effects of liquid handling robot pipetting speed on yeast growth and gene expression using growth assays and RNA-seq

**DOI:** 10.17912/micropub.biology.001566

**Published:** 2025-05-13

**Authors:** Shodai Taguchi, Ryosuke Matsuzawa, Yasuyuki Suda, Kenji Irie, Haruka Ozaki

**Affiliations:** 1 Ph.D. Program in Humanics, School of Integrative and Global Majors, University of Tsukuba, Tsukuba, Ibaraki, Japan; 2 Department of Molecular Cell Biology, Institute of Medicine, University of Tsukuba, Tsukuba, Ibaraki, Japan; 3 Bioinformatics Laboratory, Institute of Medicine, University of Tsukuba, Tsukuba, Ibaraki, Japan; 4 Doctoral Program in Medical Sciences, Degree Programs in Comprehensive Human Sciences, Graduate School of Comprehensive Human Sciences, University of Tsukuba, Tsukuba, Ibaraki, Japan; 5 Center for Artificial Intelligence Research, University of Tsukuba, Tsukuba, Ibaraki, Japan; 6 Laboratory for AI Biology, RIKEN Center for Biosystems Dynamics Research, Kobe, Hyōgo, Japan

## Abstract

Assessing the impact of experimental parameters like pipetting speed is essential in life science research but challenging in manual experiments. Recent advancements in laboratory automation enable precise control and systematic evaluation of these parameters. Here, we employed the Opentrons OT-2, an affordable, open-source liquid handling robot, to systematically investigate the effect of pipetting speed on growth and gene expression in the budding yeast *Saccharomyces cerevisiae* . Growth assays revealed no significant differences across the tested pipetting speeds (ANOVA, p > 0.05). RNA-seq analysis corroborated these findings, demonstrating highly similar gene expression profiles across all 24 samples (minimum Pearson correlation coefficient = 0.9528), with no differentially expressed genes identified by generalized linear model analysis (false discovery rate > 0.01). Our results highlight the utility of robotic platforms in optimizing experimental parameters, improving reproducibility, and enhancing accuracy in biological research, thus providing valuable insights for future applications.

**Figure 1. Pipetting speed does not affect growth rate and gene expression profile:**
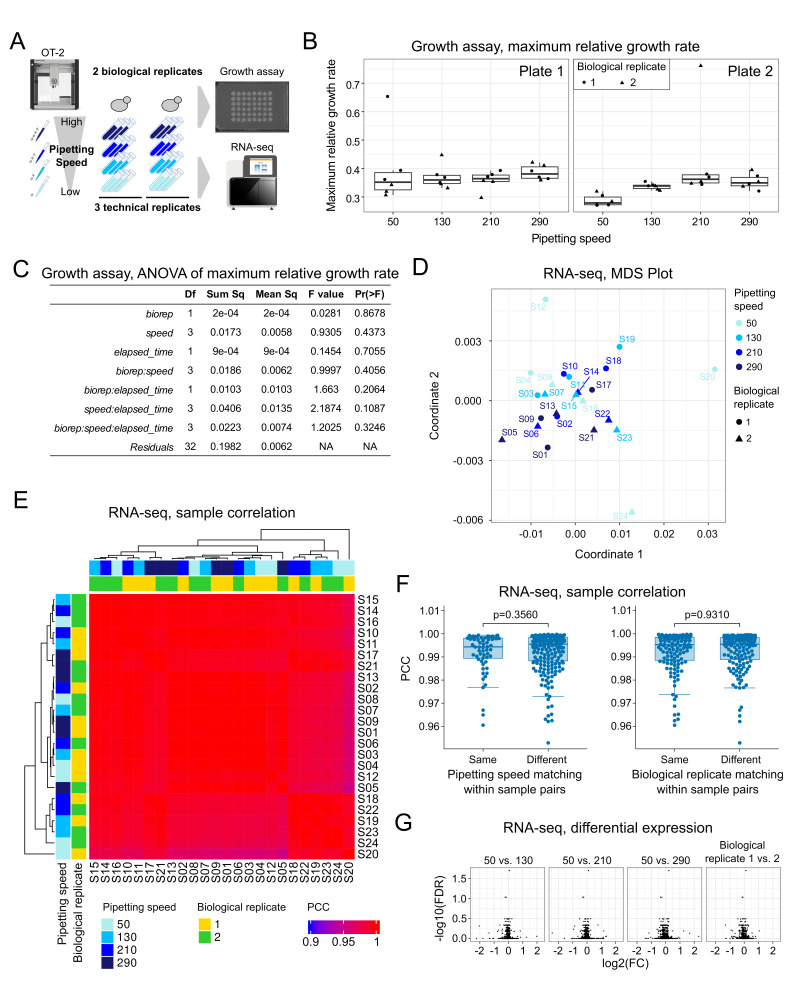
A: Schematic overview of experimental design. B: Box plots of maximum relative growth rate (maximum slope of log-scaled growth curve) for different pipetting speeds in each plate. The point shapes represent biological replicates. C: Summary of three-way ANOVA assessing the effects of biological replicate (biorep), pipetting speed, and elapsed time, along with their interactions, on the measured response variable. D: Multidimensional scaling (MDS) plot based on expression of 5,193 genes measured by RNA-seq across 24 samples. Each point represents a sample, colored by pipetting speed (µL/s), and shaped by biological replicate (circle:Replicate1, triangle: Replicate2). E: Heatmap of pairwise Pearson correlation coefficients (PCCs) of 5,193 gene expression between 24 samples. Color intensity reflects similarity in gene expression (red = high correlation, blue = low correlation). Dendrograms represent hierarchical clustering of samples. Pipetting speed and biological replicate are annotated by color keys along the margins. The PCC values are shown in Supplementary Table 1. F: Box plots showing PCC distributions for all sample pairs, grouped according to whether the paired samples shared the same pipetting speed (left) or biological replicate (right). No significant differences in PCC distributions were observed (p = 0.3560, left; p = 0.9310, right; Wilcoxon rank-sum test). G: Volcano plots of differential gene expression in the pipetting speed conditions of 130, 210, and 290 µL/sec compared to that of 50 µL/sec, and biological replicate 1 compared to biological replicate 2. Each point represents a gene. The x- and y-axes represent log2 fold change and −log10 false discovery rate (FDR), respectively.

## Description

In life science experiments, it is crucial to evaluate how various experimental parameters influence cellular responses. However, in manual experiments, certain experimental parameters are difficult to control precisely, making it challenging to determine appropriate parameter ranges. Recent advancements in laboratory automation, particularly robotic execution of experimental procedures, provide an opportunity to achieve precise control over these parameters and assess their impact. For example, an automated robotic culture system was used to precisely manipulate environmental parameters such as fluidics, optical density, temperature, and stirring rate, and to accurately measure the resulting biological responses (Wong et al. 2018) . Likewise, previous studies have demonstrated the effectiveness of automated experimental machines in determining feasible ranges of experimental parameters (Kanda et al. 2022; Sandu et al. 2024; LeSaout et al. 2016) .

One critical experimental parameter that has not been extensively studied is pipetting speed. Pipetting is a fundamental laboratory procedure used for mixing and suspending liquid reagents or cell cultures. In robotic experiments, optimizing pipetting speed is essential, representing a balance between enhancing operational efficiency and minimizing unintended effects on the cellular state (Quijano Velasco et al. 2024) . Faster pipetting speeds can generate higher shear stress at the pipette tip, which may impose mechanical forces on cells. Previous work has shown that such bioprocess forces—arising from pipetting or capillary transfer—can influence cellular behavior, including survival and differentiation (Brindley et al. 2011) . However, despite its potential importance, the influence of pipetting speed on biological experiments, —particularly when systematically varied using liquid-handling robots and evaluated through gene expression and cell growth—remains poorly investigated.

In this study, we systematically varied pipetting speed and evaluated its effects on the growth and gene expression of *Saccharomyces cerevisiae* , a widely used eukaryotic model organism in yeast research. The experiments were conducted using Opentrons OT-2, an affordable liquid-handling robot (Tegally et al. 2020) that has been commonly adopted to automate experimental procedures in yeast research (Taguchi et al. 2023; Miettinen et al. 2022; Dubencovs et al. 2021; Jiang et al. 2020; Bertaux et al. 2022; Liu, Gygi, and Paulo 2021) .

To investigate the effects of pipetting speed on cellular responses, we designed an experiment using an OT-2 liquid handling robot ( **Figure 1A** ), enabling precise control and standardization of pipetting parameters. Yeast cultures were prepared and subjected to a series of pipetting actions (aspiration, mixing, and dispensing) at the four distinct speeds (50, 130, 210, 290 μL/s). Two biological replicates, consisting of identical clones from separate culture batches, were used, and each experiment was performed in triplicate (3 technical replicates). To evaluate the impact of pipetting speed on yeast growth, a quantitative growth assay was conducted on two agar plates. In parallel, RNA sequencing (RNA-seq) was performed on the cells following pipetting to assess changes in gene expression.

In the quantitative growth assay, we performed time-lapse imaging of yeast colonies on agar plates and quantified the maximum slope of log-scaled growth curve as an estimate of maximum relative growth rates. The observed maximum relative growth rates are mostly below 0.40 h ^-1^ , which aligns with previously reported steady-state growth rates for yeast (Boender et al. 2009) , indicating that the experimental setup itself does not affect growth and allowing for valid comparisons of pipetting speed effects across different yeast experiments ( **Figure 1B** ). The analysis of variance (ANOVA) indicated that varying pipetting speeds did not significantly affect the maximum relative growth rate ( **Figure 1C** ).

Next, to investigate whether pipetting speed affects gene expression, we performed RNA-seq on yeast samples collected after pipetting at four different speeds. In total, 24 samples were analyzed. Multidimensional scaling (MDS) analysis revealed that all samples clustered closely together, with no distinct separation based on pipetting speeds or biological replicates ( **Figure 1D** ). Similarly, the Pearson correlation coefficients (PCC) of gene expression between samples were consistently high across all pairs (minimum PCC = 0.9528), indicating minimal variability due to pipetting speed or biological replicates ( **Figure 1E** ).

To provide additional perspective on the high PCC values observed, we evaluated whether pipetting speed had any discernible effect on gene expression similarity. We stratified all sample pairs into groups based on whether the pair had the same or different pipetting speeds and compared the distributions of their PCCs. If pipetting speed introduced a meaningful difference in gene expression, we would expect lower PCCs in pairs with different speeds. However, the distributions were not significantly different (p = 0.3560, Wilcoxon rank-sum test,), suggesting that pipetting speed did not impact overall gene expression similarity ( **Figure 1F, left** ). As an internal control we also compared PCC distributions between pairs of the same or different biological replicates and found no significant difference (p = 0.9310, Wilcoxon rank-sum test) ( **Figure 1F, right** ). These results support that the gene expression profiles remain stable across the pipetting speed conditions tested.

Moreover, a generalized linear model (GLM) analysis comparing gene expression across different pipetting speeds (50 vs. 130, 210, and 290 μL/s) and between biological replicates detected no differentially expressed genes with a false discovery rate (FDR) < 0.01 ( **Figure 1G** ). These results indicate that within the tested range, pipetting speed does not affect yeast gene expression profiles.

In conclusion, within the range of pipetting speeds investigated, variations in pipetting speed did not impact the maximum relative growth rate and the gene expression profiles of yeast. This suggests, in the absence of other constraints **,** the fastest possible pipetting speed (290 μL/s) can be set in yeast experiments using OT-2 and typical electronic micropipettes (for instance, the maximum pipetting speed of the Eppendorf electronic pipette Xplorer is 300 μL/0.9 s = 333.3 μL/s (“Eppendorf Liquid Handling Operating Manual Xplorer/Xplorer plus,” n.d.) . While it is uncertain whether these findings can be generalized to different conditions, such as variations in tip shape, cell density, strain, and liquid medium viscosity, we provide detailed experimental protocols and code (see Methods) to support researchers in exploring the biological impact of pipetting speed using a similar approach.

For successful robotic experiments in life sciences, it is necessary to step-by-step verify the relationship between robotic operational parameters—such as pipetting speed, plate transportation speed, and the speed of opening and closing an incubator door—and cellular responses. This study serves as a reference for determining appropriate parameter ranges in the implementation of future automated experiments.

## Methods

***Code availability***

The Python scripts, shell scripts, and Jupyter notebooks that were used in the experiments and the data analyses are available from the GitHub repository: https://github.com/bioinfo-tsukuba/OT2_pipspd_paper

***Preparation of YPD liquid medium for spotting***

Powdered reagents at a final concentration of 2% Peptone (Bacto Peptone, 211677; Thermo Fisher Scientific, Waltham, Massachusetts, US), 1% Yeast Extract (Bacto Yeast Extract, 212750; Thermo Fisher Scientific), and 0.003% Adenine (6-Aminopurine, 012-11512; FUJIFILM Wako Pure Chemical Corporation, Tokyo, Japan) were added to a 500 mL glass bottle and dissolved in 450 mL of deionized water to make up the yeast extract peptone (YP) liquid medium. In parallel, 500 mL of 20% dextrose (D(+)-glucose, 045-31167; FUJIFILM Wako Pure Chemical Corporation) solution was prepared with deionized water in a 500 mL glass bottle. The opening of each glass bottle was covered with aluminum foil, and it was autoclaved with an autoclave machine (LSX-500; TOMY SEIKO Co. Ltd., Tokyo, Japan) at 120°C for 2 h. After autoclaving, 50 mL of the 20% dextrose solution was added to 450 mL of the YP liquid medium to make up the yeast extract peptone dextrose (YPD) liquid medium.

***Preparation of YPD agar plates***

For the YPD agar medium, powdered reagents at a final concentration of 2% Peptone, 1% Yeast Extract, 2% Agar (STAR Agar L-grade 01, RSU-AL01-500G; Rikaken Co., Tokyo, Japan), and 0.003% Adenine were added to a round flask and dissolved in 450 mL of deionized water. The flask's opening was covered with aluminum foil, and it was autoclaved with an autoclave machine (LSX-500; TOMY SEIKO Co., Ltd.) at 120°C for 2 h. After autoclaving, 50 mL of 20% dextrose solution was added to 450 mL of the medium to make up the YPD agar medium. Then, the YPD agar medium was poured onto microplate petri dishes (4846-MPS, Watson Co., Ltd., Tokyo, Japan) on a clean bench. The petri dishes were left on a clean bench for 2 d to allow the agar to harden and the surface to dry.

***Yeast strains***

We used budding yeast *Saccharomyces cerevisiae* for a eukaryotic model organism in yeast. The following yeast strain was used in this study: W303-1B wild-type (WT) strain ( *MATa ade2 trp1 can1 leu2 his3 ura3* ).

***Preparation of the yeast strains***

The yeast strain from -80°C freeze stock was inoculated on the YPD agar medium and was incubated in a 30°C incubator (MIR-154-PJ; PHC Holdings Corporation) for 2 d.

***Liquid culturing***

In two autoclaved test tubes, 3 mL of the YPD liquid medium were added to each tube, and the yeast colonies were inoculated. The test tubes were incubated in a shaking water bath (Personal-11, 0069409-000; TAITEC CORPORATION, Saitama, Japan) at 30°C and 160 rpm for 16 h. Then, 3 mL of culture medium in the test tubes were inoculated in 27 mL of fresh YPD liquid medium in new 250 mL Erlenmeyer Flasks (Corning Inc. NY, US), and they were incubated in a shaker (BR-40LF, TAITEC CORPORATION) at 30°C and 160 rpm for 3 h.

***Liquid-handling robot***

The OT-2 (999-00047, Opentrons Labworks Inc.) was used. The OT-2 Single-Channel Pipette P20 GEN2 (999-00002, Opentrons Labworks Inc.) and OT-2 Single-Channel Pipette P20 GEN2 (999-00003, Opentrons Labworks Inc.) were set.

***Setting labware on the decks of the Opentrons OT-2***

We placed labware on the OT-2 decks as follows: Deck 4, Opentrons tiprack with OT-2 300 µL tips (999-00009, Opentrons Labworks Inc.); Deck 5, Opentrons tiprack with OT-2 20 µL tips (999-00007, Opentrons Labworks Inc.); Deck 6, YPD agar in Microplate Petri Dish (Watson Co., Ltd., Tokyo, Japan); Deck 7, Opentrons Tube Rack with a tube holder top for 10 tubes (999-00030, Opentrons Labworks Inc.) containing three 50 mL tubes (2324-050N; IWAKI (AGC), Shizuoka, Japan)) in A4 (30 mL culture of biological replicates #1), B4 (30 mL culture of biological replicates #2), and B3 (50 mL Milli-Q water); Deck 8, Opentrons Tube Rack with a tube holder top for 24 microtubes (999-00030, Opentrons Labworks Inc.) containing 24 x 2 mL screw cap microtubes (72.694.305; SARSTEDT K.K., Tokyo, Japan); Deck 9, YPD agar in Microplate Petri Dish (Watson Co., Ltd.); Deck 10-11, PlateShuttle (a DIY plate transportation system, https://github.com/bioinfo-tsukuba/PlateShuttle); Deck 11, 96-well polypropylene microplate (3997; Corning Inc.) was placed on the PlateShuttle ( https://github.com/bioinfo-tsukuba/PlateShuttle ). PlateShuttle is an open-source hardware/software solution for plate transportation and absorbance measurement, enabling the seamless loading/unloading of a plate into Absorbance 96 Plate Reader (Byonoy GmbH, Hamburg, Germany) on OT-2 and the measurement of absorbance. The detailed deck design is available on GitHub ( Experimental Design.pdf ).

***Execution of experiments with OT-2***

The protocol was executed using a laptop computer (MacBook Air, Apple Inc., CA, US) connected to the OT-2 via Secure Shell (SSH) and the PlateShuttle via a serial connection. Before running the experiment, we made a .env file in the ‘OT-2_pipspd_paper’ directory and filled in the path of the SSH key and IP address of the devices. We executed the Jupyter notebook ‘ run_experiment.ipynb ’ step-by-step in the Visual Studio Code. The Jupyter notebook consists of the following steps: (1) configuration of agar weight and inserting position of a microplate, (2) loading of necessary modules, (3) preparation of directories and check connection with OT-2, (4) check of connection with PlateShuttle, (5) run of ‘ run_first_half.py ’ (see below), 6) run of PlateShuttle and measurement of the absorbance at 620 nm using Absorbance 96 Plate Reader, (7) calculation of liquid volume based on absorbance at 620 nm for normalizing the cell densities between samples, and (8) run of ‘ run_second_half.py ’ (see below).

The ‘ run_first_half.py ’ script is an OT-2 protocol written in Python for pipetting yeast liquid culture from two biological replicates in 50 mL tubes on deck 7 into a 96-well plate on deck 11 for absorbance measurements, using the four different pipetting speeds. The ‘ run_second_half.py ’ script contains the OT-2 protocol for the following experimental procedures: (1) Dispensing Milli-Q water into 2 mL tubes on deck 8 and spotting wells based on dilution rates calculated from the measured absorbance, (2) Transferring yeast from each sample tube into the 2 mL tubes at specified pipetting speeds, (3) Dispensing yeast from the 2 mL tubes into the spotting wells at specified pipetting speeds, and (4) Spotting each sample onto agar plates on deck 6 and deck 9.

***Pipetting speed setting***

Since information on the possible range of pipetting speeds of OT-2 micropipettes is not officially provided, we determined its maximum and minimum pipetting speeds experimentally. Specifically, we measured the total elapsed time for performing a mixing task 10 times while varying the pipetting speed. We observed that the total elapsed time plateaued at a maximum speed of 290 µL/sec and a minimum speed of 50 µL/sec. Thus, we determined that the maximum pipetting speed of the OT-2 is 290 µL/sec and the minimum is 50 µL/sec. By taking values at equal intervals between the minimum and maximum values, we considered an arithmetic progression and set the pipetting speed levels to 50, 130, 210, and 290 µL/sec.

***The order of spotting***

As we used single-channel pipettes in the OT-2 experiment, only one spot could be processed at a time, meaning that the order of spotting determines the differences in spotting time among colonies. Since the average doubling time of yeast is 90 minutes and the time difference between first spotted colony and last spotted one is about 70 minutes, colonies spotted earlier may appear to reach the growth curve's onset and maximum slope earlier. Thus, in the spot assay conducted with ‘ run_second_half.py ’, we specified the order of spotting to prevent confounding between pipetting speed and spotting order ( agar_plate_annotation.csv , colonies were spotted from top to bottom).

***The positions of spots***

In preliminary experiments, when spots were arranged in a 4×6 matrix on each plate, the perimeter spots exhibited faster growth rates than the inner spots. This difference is likely due to a stronger inhibitory effect when more adjacent colonies are present (Palková et al. 1997) . To minimize growth rate differences caused by spot positions in the spot assay conducted with ‘ run_second_half.py ’ additional spots were placed outside the perimeter, expanding the matrix from 4×6 to 6×8. This ensured that the 4×6 spots used for growth data were all positioned as inner spots on each plate. The spot arrangement design is available on GitHub ( Experimental Design.pdf ).

***Quantitative cell growth assay***

Upon completing the OT-2 experiments, the microplate Petri dishes were covered with lids and placed agar-side down on a flatbed scanner (GT-X980, EPSON, Tokyo, Japan) inside the incubator, ensuring proper alignment with the scanner corners. The scanner was connected to a computer running Ubuntu 20.04 via a USB serial connection through the incubator vent. Time-lapse scanning was initiated using a custom script ( scan-epson-lower.sh ), which executed the Linux command ‘scanimage’ to capture images at 600 dpi resolution every 10 minutes for 72 hours, yielding a total of 432 images.

For subsequent image processing, a dedicated script was used to crop individual plates from each image and rotate them for grid-aligned spot positioning ( 001_rotate-crop.ipynb ). Cell density was quantified from the processed images using baQFA (version 0.1.0) (Frey et al. 2021) . The maximum relative growth rate, defined as the ‘nr’ parameter in baQFA, corresponds to the maximum slope of log-scaled growth curve of cell density. In baQFA, the ‘nr’ parameter (h ^−1^ ), a numerical estimate of the intrinsic growth rate, was calculated using the following steps: (1) Fitting locally weighted scatterplot smoothing (LOESS) functions to the log-transformed growth curve data, (2) Dividing the domain into intervals of width 10 ^−4^ , (3) Calculating the slope for each interval, (4) Selecting the maximum slope among all intervals. The baQFA analysis codes and results are available on GitHub ( 240415_baQFA ).

***RNA-Seq sample preparation***

Numbers 1–24 were labeled on the screw caps and 2 mL tubes. Cells were collected by centrifugation at 10,000 × g for 1 minute (MX-305; TOMY, Tokyo, Japan). Each 2 mL tube was then supplemented with 1.00 g of 0.5 mm Zirconia/Silica Beads (11079105z; BioSpec Products, Inc., Oklahoma, US) and 500 µL of ISOGEN (319-90211; Nippon Gene Material Co., Ltd., Tokyo, Japan). Cells were homogenized using a Micro Smash (MS-100R; TOMY) at 3,500 rpm for 240 seconds, followed by the addition of an additional 500 µL of ISOGEN. Homogenization was repeated at 3,500 rpm for 60 seconds. Finally, 800 µL of the supernatant was collected into a new 1.5 mL tube for RNA-Seq library preparation.

***RNA-Seq***

RNA sequencing was performed by Tsukuba i-Laboratory LLP (Tsukuba, Japan) as follows. RNA quantification and quality assessment were conducted using the Bioanalyzer RNA 6000 Pico Kit (5067-1513; Agilent Technologies). The RNA integrity number (RIN) (Schroeder et al. 2006) was confirmed to be greater than 7.60.

For RNA-seq, poly(A) RNA was purified from 100 ng of total RNA using a Poly-T column kit (E7490, New England Biolabs (NEB)). RNA-seq libraries were prepared from the purified RNA using the NEBNext Ultra Directional RNA Library Prep Kit for Illumina (E7420; NEB), following the manufacturer’s protocol. Finally, 1.5 pM of pooled library DNA was sequenced (36 nt paired-end reads) on an Illumina NextSeq 500 System (SY-415-1001; Illumina) with the NextSeq 500 High-Output v2 Kit (FC-505-2005; Illumina).

***RNA-seq data preprocessing***

The reference genome ( W303_SGD_2015_JRIU00000000.fsa.gz ) and gene annotation file ( W303_JRIU00000000_SGD.gff.gz ) for *Saccharomyces cerevisiae* W303 were downloaded from the Saccharomyces Genome Database ( http://sgd-archive.yeastgenome.org/sequence/strains/W303/W303_SGD_2015_JRIU00000000/ ). We note that the genome and gene annotation files are accessible via an HTTP (not HTTPS) URL provided above. Depending on web browser security settings, downloads initiated via HTTP may trigger security warnings or be temporarily blocked. Users encountering download issues should explicitly allow or bypass the browser warnings to successfully retrieve the genome files.

FASTQ reads were merged, and adapter sequences and low-quality bases were trimmed using fastp (version 0.23.2) with the following parameters: '-q 15 -n 10 -t 1 -T 1 -l 20 -w 16 -A'. The trimmed reads were then aligned to the *S. cerevisiae* W303 reference genome using STAR (version 2.7.10a) with the parameters '--quantMode TranscriptomeSAM --outSAMtype BAM SortedByCoordinate'. Read counts and transcript abundance (TPM, Transcripts Per Million) were quantified using RSEM (version 1.3.1) with the parameters 'rsem-calculate-expression --alignments --paired-end --strandedness reverse --estimate-rspd'.

The RNA-seq data from this study have been deposited in NCBI's Gene Expression Omnibus (GEO) (Edgar, Domrachev, and Lash 2002) and available under the accession number GSE266380 ( https://www.ncbi.nlm.nih.gov/geo/query/acc.cgi?acc=GSE266380 ).

***Statistical analysis***

We performed ANOVA using the ‘anova’ function in R (version 4.4.2) to assess the effects of pipetting speed (speed), biological replicates (biorep), and elapsed time (elapsed_time) on the maximum relative growth rate (nr; the maximum slope of the log-scaled growth curve). Elapsed time for each yeast spot was calculated from OT-2 experimental logs, measuring the duration from when the yeast was taken from the liquid culture in 50 mL tubes on deck 7 to when the yeast was spotted. Nr was normalized with the median of nr within the same plate. The model for ANOVA was as follows: nr ~ biorep + speed + elapsed_time + biorep * speed + speed * elapsed_time + elapsed_time * biorep + biorep * speed * elapsed_time. The source code and ANOVA results are available on GitHub ( integrated_analysis.md ).

RNA-seq data analysis was conducted using R (version 4.4.0). Lowly expressed genes (average count ≤ 1 across samples) were excluded. The effects of biological replicates and pipetting speeds were analyzed using the GLM mode of EdgeR (version 4.1.31). The model for GLM was as follows: count ~ speed + biorep. False discovery rates (FDRs) were computed using the Benjamini-Hochberg procedure for multiple testing correction. Data visualization was performed with ggplot2 (version 3.5.1). Source code and result files are available on GitHub ( analysis.md ).

***Declaration of generative AI and AI-assisted technologies in the writing process***

During manuscript preparation, the authors used ChatGPT (GPT-4o) (OpenAI, CA, USA), a large language model, for English translation, grammar correction, and stylistic refinement. The AI tool was applied to individual sentences and paragraphs but was not used to generate new content or modify scientific conclusions. All content was thoroughly reviewed and edited by the authors, who take full responsibility for the manuscript's accuracy, originality, and integrity.

## Reagents

**Table:** 

STRAIN	GENOTYPE	AVAILABLE FROM
W303-1B wild-type	*MATa ade2 trp1 can1 leu2 his3 ura3*	(Tadauchi et al., 2001)

**Table:** 

REAGENT	PRODUCT ID	AVAILABLE FROM
Bacto Peptone	211677	Thermo Fisher Scientific
Bacto Yeast Extract	212750	Thermo Fisher Scientific
Adenine	012-11512	FUJIFILM Wako Pure Chemical Corporation
D(+)-glucose	045-31167	FUJIFILM Wako Pure Chemical Corporation
STAR Agar L-grade 01	RSU-AL01-500G	RIKAKEN HOLDINGS CO., LTD.
ISOGEN	319-90211	Nippon Gene Material Co.,Ltd.
Bioanalyzer RNA 6000 Pico Kit	5067-1513	Agilent Technologies, Inc.
Poly-T column kit	E7490	New England Biolabs
NEBNext Ultra Directional RNA Library Prep Kit	E7420	New England Biolabs

**Table:** 

Material	PRODUCT ID	AVAILABLE FROM
OT-2 20µL Tips	999-00007	Opentrons Labworks Inc.
OT-2 300µL Tips	999-00009	Opentrons Labworks Inc.
Screw cap micro tube, 2 ml	72.694	Sarstedt K.K.
4-in-1 Tube Rack Set	999-00030	Opentrons Labworks Inc.
Microplate Petri Dish with Lid Sterilized	4846-MPS	Watson Co., Ltd., Tokyo, Japan
Corning 250 mL Polycarbonate Erlenmeyer Flask with Vent Cap	431144	Corning Inc. NY, US
96-well polypropylene microplate	3997	Corning Inc. NY, US

## Data Availability

Description: Supplementary Table 1: Pairwise Pearson correlation coefficients (PCCs) of 5,193 gene expression between 24 samples.. Resource Type: Dataset. DOI: https://doi.org/10.22002/82xv5-wk067
